# Serum TNFRII: A promising biomarker for predicting the risk of subcentimetre lung adenocarcinoma

**DOI:** 10.1111/jcmm.15071

**Published:** 2020-02-19

**Authors:** Minjuan Hu, Yanwei Zhang, Beibei Sun, Yuqing Lou, Xueyan Zhang, Huimin Wang, Chengya Huang, Wei Zhang, Tianqing Chu, Baohui Han

**Affiliations:** ^1^ Department of Pulmonary Medicine Shanghai Chest Hospital Shanghai Jiao Tong University Shanghai China; ^2^ Department of Central Laboratory Shanghai Chest Hospital Shanghai Jiao Tong University Shanghai China; ^3^ Department of Anesthesiology Shanghai Chest Hospital Shanghai Jiao Tong University Shanghai China

**Keywords:** biomarker, risk, serum TNFRII, subcentimetre lung adenocarcinoma

## Abstract

Early diagnosis of lung adenocarcinoma requires effective risk predictors. TNFRII was reported to be related to tumorigenesis, but remained unclear in lung cancer. This research set out to investigate the relationship between the sTNFRII (serum TNFRII) level and the risk of lung adenocarcinoma less than 1 cm in diameter. Seventy‐one pairs of subcentimetre lung adenocarcinoma patients and healthy controls were analysed through multiplex bead‐based Luminex assay and found a significantly lower expression of sTNFRII in patients with subcentimetre lung adenocarcinoma than that in the healthy controls (*P* < .001), which was further verified through ONCOMINE database analysis. Increased levels of sTNFRII reduced the risk of subcentimetre lung adenocarcinoma by 89% (*P* < .001). Patients with a higher level of BLC had a 2.70‐fold (*P* < .01) higher risk of subcentimetre adenocarcinoma. Furthermore, a higher BLC/TNFRII ratio was related to a 35‐fold higher risk of subcentimetre adenocarcinoma. TNFRII showed good specificity, sensitivity and accuracy (0.72, 0.75 and 0.73, respectively), with an AUC of 0.73 (*P* < .001). In conclusion, the present study assessed the value of sTNFRII as a potential biomarker to predict the risk of subcentimetre lung adenocarcinoma and provided evidence for the further use of TNFRII as an auxiliary marker in the diagnosis of subcentimetre lung adenocarcinoma.

## 1 INTRODUCTION

The latest epidemiological investigation of lung cancer covering 185 countries and 18 million people showed that the incidence and mortality of lung cancer were 11.6% and 18.4%, respectively, both of which were the highest among all malignancies.^1^ Annual low‐dose computed tomography (LDCT) scanning to detect early‐stage lung cancer in the high‐risk population was recommended by the USPSTF (US Preventative Services Task Force) in 2013,^2^ and screening of lung cancer by LDCT is also gradually being carried out in China.^3^ Owing to advances in imaging techniques such as CT, LDCT and 3D imaging, the detection rate of subcentimetre lung cancer, of which adenocarcinoma accounts for the majority, is increasing remarkably.^3^, ^4^ Meanwhile, Kato et al^5^ found that 54.44% of subcentimetre adenocarcinomas were invasive. Small‐sized and low‐density nodules are difficult to access by bronchial biopsy and percutaneous lung biopsy, making a safe diagnosis and further treatment difficult.^6^ In addition, evidence has shown that imaging cannot be a stand‐alone method for the diagnosis of early‐stage lung adenocarcinoma either,^6^, ^7^ and biomarkers are urgently needed.

Considerable evidence has indicated the role of chronic inflammation in tumorigenesis,^8^, ^9^ which remains unclear in lung cancer. Recent established inflammatory factors such as IL‐8, C‐reactive protein (CRP), pentraxin 3 (PTX3) and tumour necrosis factor receptor‐2 (TNFRII) have been linked to increased lung cancer risk.^10^, ^11^, ^12^, ^13^ Our prior study of 10 widely evaluated inflammatory biomarkers found that elevated levels of B lymphocyte chemoattractant (BLC) increased the risk of subcentimetre lung adenocarcinoma by 2.90‐fold.^14^

It has now been well‐established from a variety of studies that the TNF/TNFR superfamily is correlated with the tumorigenesis and clinical efficacy of multiple tumours, including NSCLC.^11^, ^12^, ^15^, ^16^ Serum TNFRII is considered to be a hallmark of tumorigenesis because it has been proven to positively regulate cell growth through activating the NF‐κB pathway.^17^, ^18^ Therefore, this study aimed to analyse the levels of CRP, TNFRII and BLC in 71 patients with subcentimetre lung adenocarcinoma and 71 healthy controls to further evaluate the efficacy of sTNFRII in predicting the risk of subcentimetre lung adenocarcinoma.

## 2 MATERIALS AND METHODS

### 2.1 Patients

Patients who were radiographically diagnosed with subcentimetre lung nodules (the tumour size was less than 1 cm) confirmed as primary lung adenocarcinoma by post‐operative pathology at the Shanghai Chest Hospital were screened. The following entry criteria were applied: (a) patients radiographically and pathologically proven to have subcentimetre lung adenocarcinoma, (b) obtainable pre‐operative blood samples, and (c) patients gave their informed consent before enrolment. The exclusion criteria included the following: (a) a lack of epidemiologic data, (b) a lack of consent for aetiological studies, (c) patients with any signs of infection or obstructive pneumonia to minimize the effects of concomitant inflammatory states on measured cytokines. Healthy control subjects were recruited from the Huadong Sanatorium, with each healthy control matching one patient's age (±2 years), gender and smoking history.

### 2.2 Serum biomarkers

Blood samples were drawn before treatment and stored in numbered heparin tubes. The supernatant was then collected after centrifugation and stored at −80°C. To standardize clotting conditions, all sera were separated within 2 hours after blood samples were obtained. A multiplex bead‐based Luminex assay was applied to measure the concentrations of CRP, TNFRII and BLC with reference to a standard curve with four or five parameters. Samples from patient cases and healthy subjects were placed in adjacent wells of the same batch for full control, and each serum sample was measured twice with the average used for subsequent analysis. The serum biomarkers were measured in pooled serum samples, and a pair of blind duplicate samples from each batch was used to assess drift across batches and reproducibility.

### 2.3 Statistical analysis

Comparisons between the two groups were carried out using Student's *t* test, Pearson's chi‐square test, Fisher's exact test or Wilcoxon's rank‐sum test as appropriate. The OR (odds ratio) and 95% CI (confidence interval), which reflect the strength of the correlation between each marker with subcentimetre lung adenocarcinoma risk, were calculated by conditional logistic regression. In addition to the variables used for matching, the history of cardiovascular diseases, history of chronic obstructive pulmonary disease, frequency of aspirin use and family history of lung cancer were also included in the analysis.

We used ROC curve analysis to assess the efficacy of inflammatory molecules in the diagnosis of subcentimetre adenocarcinoma. The best cut‐off point for ROC analysis was obtained by screening the value with the maximum sum of the sensitivity and specificity.

The ONCOMINE database (http://www.oncomine.org), which is a database of publicly available cancer microarray data, was used to compare the TNFRII mRNA levels between normal tissues and cancer specimens in four datasets. A fold change over 2 was defined as significant, and the *P* value was obtained using the Student's *t* test.

All statistically significant tests were two‐sided. A difference with a *P* value < .05 was considered to be significant. Data management and analysis were performed using SPSS 20.0.

## 3 RESULTS

### 3.1 Basic characteristics

**Figure 1:**
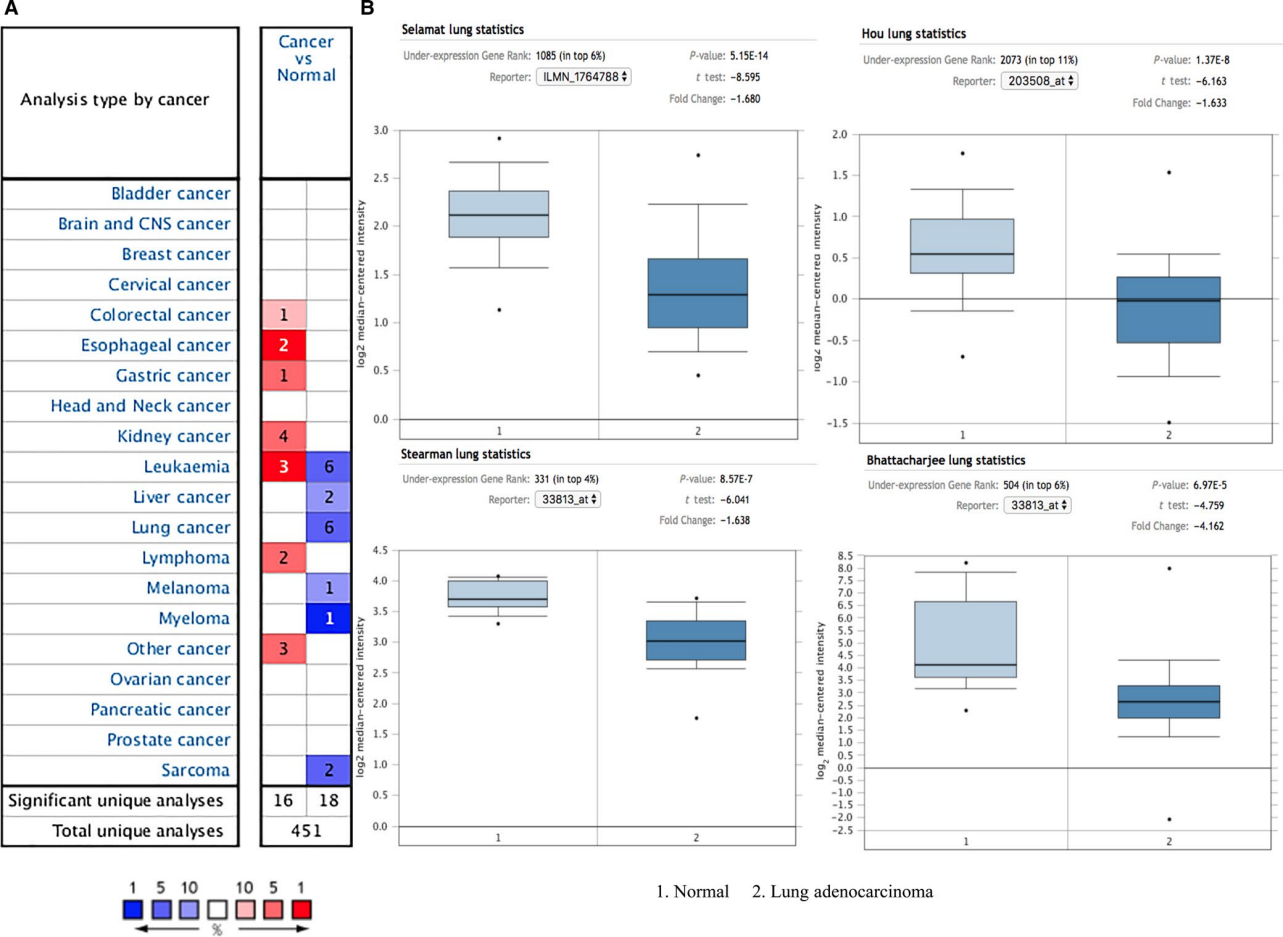
Comparison of mRNA levels of TNFRII in tumour and normal tissue based on ONCOMINE database. A, Down‐regulation of TNFRII was found in 6 cancer types. B, The levels of TNFRII mRNA were significantly decreased from normal lung tissue to lung adenocarcinoma in four datasets. *P* value was calculated by the student's *t* test

The initial samples were from 71 subcentimetre lung adenocarcinoma patients, and 71 healthy controls matched for age, gender and smoking status (Table 1). Of these 71 patients, 52 (73.20%) were women, and 19 (26.80%) were men, with a mean (standard deviation or SD) age of 56.01 (8.91) years. Patients without a history of smoking accounted for 85.96% (57/71) of the patients. As shown in the data in Table 1, no significant differences in gender, age, smoking status, CRP levels or BLC levels between patients and controls were evident. However, a decrease in sTNFRII levels in patients with subcentimetre adenocarcinoma compared to those in normal controls was statistically significant (156.2 vs 244.5, respectively, *P* < .001, Table 1). Through subsequent ONCOMINE data mining, TNFRII was down‐regulated in 6 cancer types (Figure 1A). For lung cancer, we verified in four datasets that the mRNA level of TNFRII was significantly lower in patients with lung adenocarcinoma than in normal lung tissue (Figure 1B).

**Table 1:** Baseline characteristics of subcentimetre lung adenocarcinoma patients and matched controls

Characteristics	Patients (N = 71)	Controls (N = 71)	*P* value
Age (y), mean (SD)	56.01 (8.91)	55.96 (8.98)	.970
Sex, N (%)			
Female	52 (73.20)	52 (73.20)	1.000
Male	19 (26.80)	19 (26.80)	
Smoking status, N (%)			
Ever smokers	14 (14.04)	14 (14.04)	1.000
Never smokers	57(85.96)	57 (85.96)	
Biomarkers(pg/mL), mean (SD)			
CRP	1 667 952.7 (31 528.9)	1 182 699.2 (18 162.1)	.181
BLC	53.5 (31.9)	42.8 (36.6)	.066
TNFRII	156.2 (74.5)	244.5 (122.8)	7.5 × 10^−7^

Note

*P* was calculated by the *t* test or the Mann‐Whitney *U* test or the chi‐squared test where appropriate.

Abbreviations: SD, standard deviation; N (%), number (percentage).

### 3.2 Serum inflammatory factors correlate with subcentimetre lung adenocarcinoma risk

A comparison of the two groups revealed that elevated levels of TNFRII reduced the risk of subcentimetre adenocarcinoma by 89% (OR 0.11, 95% CI: 0.04‐0.30, *P* < .001, Table 2). A higher level of BLC in the serum increased the risk of subcentimetre lung adenocarcinoma by 2.70‐fold (95% CI, 1.31‐5.58, *P* < .01, Table 2) compared with that of the group with a lower level of BLC. To further assess the potential link, we examined the efficacy of the BLC/TNFRII ratio in predicting the risk of subcentimetre lung adenocarcinoma. As shown in Table 2, a higher BLC/TNFRII ratio was related to a 35‐fold elevated risk of subcentimetre adenocarcinoma compared with that in the group with a lower BLC/TNFRII ratio.

**Table 2:** Risk prediction of TNFRII and BLC for subcentimetre lung adenocarcinoma

Biomarkers, pg/mL	Patients N (%)	Controls N (%)	OR (95% CI)	*P* value
TNFRII				
<194.4	53 (74.6)	20 (28.2)	1	2.4 × 10^−5^
≥194.4	18 (25.4)	51 (71.8)	0.11 (0.04‐0.30)	
BLC				
<36.0	20 (28.2)	37 (52.1)	1	7.0 × 10^−3^
≥36.0	51 (71.8)	34 (47.9)	2.70 (1.31‐5.58)	
BLC/TNFRII				
1	24 (33.8)	58 (81.7)	1	4.5 × 10^−4^
2	47 (66.2)	13 (18.3)	35.00 (4.80‐255.47)	

Note

Adjusted for matching variables (age, gender and smoking history).

Abbreviations: 95% CI, 95% confidence interval; N (%), number (percentage); OR, odds ratio.

The association of TNFRII, BLC and BLC/TNFRII with subcentimetre lung adenocarcinoma risk was stratified by age, gender and smoking status, respectively (Table 3). Elevated TNFRII levels were found to reduce the risk in never smokers, in either females or males, and in either younger or older subcentimetre lung adenocarcinoma patients.

**Table 3:** Stratified analyses of three significant biomarkers on risk in subcentimetre lung adenocarcinoma

Biomarkers, pg/mL	Age < 60 y				Age ≥ 60 y			
	Patients, N	Controls, N	OR (95% CI)	*P*	Patients, N	Controls, N	OR (95% CI)	*P*
TNFRII: Low/High	36/10	13/32	0.12 (0.04‐0.40)	5.0 × 10^−4^	17/8	7/19	0.09 (0.01‐0.70)	.02
BLC: Low/High	10/36	20/25	2.66 (1.04‐6.81)	.04	10/15	17/9	2.50 (0.78‐7.97)	.12
BLC/TNFRII: Low/High	11/35	35/10	25.0 (3.40‐184.5)	.02	13/12	23/3	65.3 (0.31‐13 885.6)	.12

Biomarkers, pg/mL	Females				Males			
	Patients, N	Controls, N	OR (95% CI)	*P*	Patients, N	Controls, N	OR (95% CI)	*P*
TNFRII: Low/High	38/14	17/35	0.09 (0.02‐0.37)	9.2 × 10^−4^	15/4	3/16	0.14 (0.32‐0.63)	.01
BLC: Low/High	14/38	30/22	3.66 (1.49‐9.04)	4.8 × 10^−3^	6/13	7/12	1.25 (0.34‐4.65)	.74
BLC/TNFRII: Low/High	16/36	42/10	27.0 (3.67‐198.69)	1.2 × 10^−3^	8/11	16/3	65.3 (0.22‐19 220.5)‐	.15

Biomarkers, pg/mL	Never smokers				Ever smokers			
	Patients, N	Controls, N	OR (95% CI)	*P*	Patients, N	Controls, N	OR (95% CI)	*P*
TNFRII: Low/High	43/14	17/40	0.10 (0.02‐0.43)	1.9 × 10^−3^	10/4	3/11	0.17 (0.02‐1.38)	.09
BLC: Low/High	16/41	23/34	3.20 (1.17‐8.73)	.02	4/10	7/7	3.00 (0.31‐28.84)	.34
BLC/TNFRII: Low/High	18/39	49/8	65.3 (2.62‐1627.3)	.01	6/8	9/5	65.3 (0.006‐702 391.4)	.38

Note

Adjusted for matching variables (age, gender and smoking history).

Abbreviations: 95% CI, 95% confidence interval; N, number; OR, odds ratio.

### 3.3 TNFRII and BLC for subcentimetre lung adenocarcinoma diagnosis

**Figure 2:**
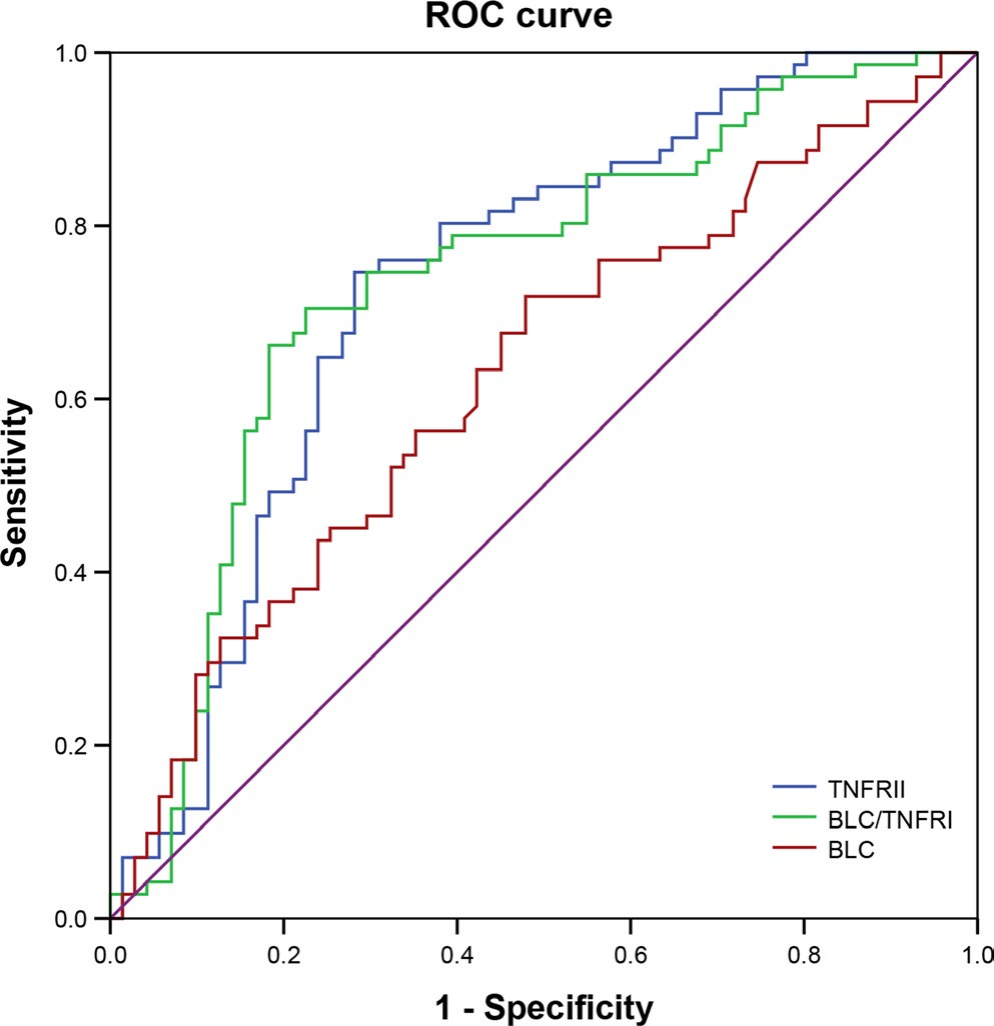
ROC curve of TNFRII, BLC and BLC/TNFRII. ROC, receiver operating characteristic. The cut‐off point was established by selecting the value with the maximum sum of sensitivity and specificity

As shown in Table 4, TNFRII, BLC and the BLC/TNFRII ratio were found to exhibit high sensitivities of 0.75, 0.72 and 0.66, respectively. High sensitivity, specificity and accuracy (0.75, 0.72 and 0.73, respectively) for TNFRII suggested its potential use as a marker for the diagnosis of subcentimetre adenocarcinoma. Further, ROC curve analysis of TNFRII showed an AUC of 0.73 ranging from 0.65 to 0.82 (*P* < .001, Table 4, Figure 2).

**Table 4:** ROC analysis of TNFRII and BLC for diagnosing subcentimeter lung adenocarcinoma

Biomarkers	Sensitivity (%)	Specificity (%)	Accuracy (%)	AUC	95% CI	*P* value
TNFRII	74.6	71.8	73.2	0.73	0.65‐0.82	2.0 × 10^−6^
BLC	71.8	52.1	62.0	0.63	0.54‐0.72	8.9 × 10^−3^
BLC/TNFRII	66.2	81.7	73.9	0.74	0.66‐0.82	1.0 × 10^−6^

Abbreviations: 95% CI, 95% confidence interval; AUC, area under curve.

## 4 DISCUSSION

To our knowledge, no study has established a credible way to estimate the risk of lung adenocarcinoma in people with subcentimetre nodules. This is the first study to evaluate serum TNFRII as an assisted biomarker for the diagnosis of very early‐stage lung adenocarcinoma in the Chinese population. A positive correlation between elevated levels of sTNFRII and an 89% reduced risk of subcentimetre lung adenocarcinoma was found in the current study. In addition, the satisfactory sensitivity, specificity and accuracy of sTNFRII suggest that it might serve as a diagnostic marker for subcentimetre lung adenocarcinoma.

Many validated inflammation indicators are linked to lung cancer risk; these include the use of corticosteroids, chronic obstructive pulmonary disease (COPD) and chronic pulmonary infection, even when smoking was ruled out.^12^, ^19^, ^20^, ^21^ Our previous study also analysed 10 widely evaluated inflammatory biomarkers and noted an independent association between BLC and significant risk for early‐stage lung adenocarcinoma.^14^ BLC, also known as BCA‐1 or CXCL13, was found to be involved in the carcinogenesis.^22^, ^23^, ^24^ In the present study, an elevated level of BLC was further shown to increase the risk of subcentimetre lung adenocarcinoma by 2.70‐fold. However, the specificity and accuracy of BLC for early‐stage diagnosis are quite limited partly due to no significant difference was found between patients and controls.

Existing research has proven increased levels of TNFRII in tumour cell lines and tumour tissues, which predicts a later clinical stage and worse prognosis,^25^, ^26^ but far too little attention has been paid to the role of TNFRII in risk prediction for early‐stage lung adenocarcinoma. TNFRII functions as a bidirectional monitor that can be immunosuppressive or immunostimulatory,^17^, ^18^, ^27^ and its soluble form can act as a ligand by binding to transmembrane TNF‐α.^28^ Immunosuppression is thought to be the dominant function of soluble TNFRII; once Treg cells are activated, they generate a large amount of soluble TNFRII.^29^

In the current study, increased sTNFRII levels were found to be significantly related to the down‐regulated risk of very early‐stage lung adenocarcinoma. A possible explanation for this might be that immune activity against tumours is enhanced, whereas immune escape has not yet occurred in early‐stage tumours. With the immunosuppressive mechanism inactivated, less sTNFRII is released by Treg cells. Collectively, not only the level of TNFRII but also its function is of great significance for the tumorigenesis of subcentimetre lung adenocarcinoma. To develop a full picture of sTNFRII, additional studies will need to be undertaken.

Our research has several advantages, including the comprehensive clinical and epidemiological information of the patients analysed. Exact matching makes the results of this prospective study applicable to clinical practice. New high‐throughput technology allows the rapid and simultaneous measurement of various serum proteins. Blood tests are easy to handle, non‐invasive and cost‐effective but still reflect the abnormal inflammatory microenvironment of the tumour.^7^

In summary, our study identified a reliable biomarker for predicting the risk of subcentimetre lung adenocarcinoma. Moreover, the serum TNFRII level may be an effective biomarker with high specificity and sensitivity for the clinical diagnosis of subcentimetre lung adenocarcinoma. Further studies that take benign nodules, other inflammatory factors and deeper mechanisms into account will provide aetiological insights and help identify individuals with the highest risk of lung adenocarcinoma.

## CONFLICT OF INTEREST

The authors have no conflicts of interest to declare.

## AUTHOR CONTRIBUTIONS

BH and YZ participated in the design of the study. BS, YL and CH carried out clinical information matching and sample collection. BS, XZ and HW performed experiments. MH, YZ, WZ and TC analysed experimental data and conducted graphs and tables. MH and YZ wrote the manuscript. All authors read and approved the final manuscript.

## ETHICAL APPROVAL

All procedures performed in studies involving human participants were in accordance with the ethical standards of the institutional and/or national research committee.

## INFORMED CONSENT

Informed consent was obtained from all individual participants included in the study.

## Data Availability

The data that support the findings of this study are available from the corresponding author upon reasonable request.
